# Calcyclin (S100A6) Attenuates Inflammatory Response and Mediates Apoptosis of Chondrocytes in Osteoarthritis *via* the PI3K/AKT Pathway

**DOI:** 10.1111/os.12990

**Published:** 2021-05-04

**Authors:** Xiao‐fei Zhang, Jian‐xiong Ma, Yuan‐lin Wang, Xin‐long Ma

**Affiliations:** ^1^ Department of Joint Surgery Tianjin Hospital Tianjin China; ^2^ Institute of Orthopaedics Tianjin Hospital Tianjin China; ^3^ Graduate School of Tianjin Medical University Tianjin Medical University Tianjin China

**Keywords:** Apoptosis, Calcyclin, Inflammation, PI3K/AKT pathway, S100A6

## Abstract

**Objective:**

To clarify the regulatory effect of Calcyclin (S100A6) on chondrocytes apoptosis and its relationship with progression of osteoarthritis in an effort to explore potential therapeutic targets for osteoarthritis.

**Method:**

Immunofluorescence assay was produced to identify the rat chondrocyte sample and western blots assay was detected the expression changes of S100A6 between control group and osteoarthritis model which induced by interleukin‐1β. Adenovirus were transfected into the chondrocytes *in vitro*, in order to regulate the S100A6 expression. The influence of S100A6 on inflammatory reaction of osteoarthritis was detected by RT‐PCR. Also, Caspase‐3 activity assay and TUNEL assay were performed to evaluate the apoptosis changes. In addition, RT‐PCR and western blots were performed to verify that S100A6 mediated the PI3K/AKT signaling pathway. Through the usage of pathway regulator, we detected S100A6 produced the effect by mediating the PI3K/AKT pathway.

**Results:**

We determined the expression of S100A6 decreased in osteoarthritis model, the relative expression level in osteoarthritis model was about 0.5 fold compared with control group. Through adenovirus transfection we revealed that the inflammatory factors of osteoarthritis (interleukin‐6 and matrix metalloproteinase‐13) showed a negative correlation with the S100A6 expression. The relative expression level of interleukin‐6 and matrix metalloproteinase‐13 were 1.534 and 1.259 when S100A6 was up‐regulated and the values were up to 2.445 and 2.074, respectively, when S100A6 was down‐regulated. Also, the data verified the apoptosis could be reduced when the S100A6 was up‐regulated and be activated when the S100A6 was down‐regulated, the Caspase‐3 activity was 16.512 U/μg and 24.45 U/μg respectively. Similar results were shown in TUNEL assay, the apoptosis index was 4.46% and 31.44%, respectively. Additionally, the results of polymerase chain reaction and western blots both demonstrated that the expression level of PI3K and AKT were increased when S100A6 was up‐regulated, conversely the expression level of those two signal modules were reduced if the S100A6 was down‐regulated. More importantly, the apoptosis triggered by S100A6 can be offset by the PI3K/AKT pathway inhibitor and activator (LY294002 and IGF‐1), the values of Caspase‐3 activity and apoptosis index became close to the untreated osteoarthritis group. The experimental results in this study were statistically significant.

**Conclusion:**

We investigated that Calcyclin (S100A6) relieved the inflammation and mediated the chondrocyte apoptosis through PI3K/AKT pathway and we confirmed that S100A6 might be an attractive therapeutic target.

## Graphical abstract

S100A6 attenuates inflammatory response and mediates apoptosis of chondrocytes via the PI3K/AKT pathway. S100A6 plays a significant role in the progression of osteoarthritis.

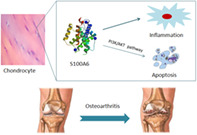

## Introduction

Osteoarthritis (OA) is one of the serious age‐related diseases, which has a high disability rate. In the environment of the aging society, the number of patients with this disease is increasing year by year. According to statistics, the prevalence rates of men and women are 10% and 18% respectively in people over 60 years old ^1^. Some scholars predict that osteoarthritis will become the fourth major disability disease in 2020^2^. OA is characterized by the progressive degradation of articular cartilage. The degradation process includes increasing loss of articular cartilage, which is closely related with chondrocyte apoptosis, and accompanied with remodeling of subchondral bone and osteophyte formation^3^. These changes bring Joint pain, deformity, limited movement and other symptoms, which seriously affect the quality of life of patients^4^, ^5^. At present, the intervention to control the cartilage degradation is limited, and the end‐stage patients inevitably need to undergo joint replacement surgery. However, the existence of various postoperative complications and joint revision operation caused by infection or loosening bring great burden to patients. Due to the lack of early diagnosis and treatment options, there is an urgent requirement to clarity the molecular mechanisms of cartilage degeneration in order to explore the novel biomarkers or therapeutic targets.

Calcyclin, which is also called S100 calcium binding protein A6 (S100A6), is a member of S100 protein family and localized in cytoplasm and nucleus in many different kinds of cells^6^. It is an acidic protein which is composed of 90 amino acids and the molecular formula is C_454_H_736_N_120_O_138_S_3_. S100A6 has many secondary structures, most of which are helix, especially it has two EF hand structures at the carboxyl end and the amino terminal respectively, which can bind to calcium ions. Then the conformation of the protein changes after combination, exposing the binding site for target protein to exert biological functions^7^. The roles of S100A6 were widely discussed in the literature. Many studies have found the expression level of S100A6 associated closely with the physiological process of some cancer diseases and affect their apoptosis or proliferation activities, such as hepatocellular carcinoma, cervical cancer, and gastric cancer^8^, ^9^, ^10^. For other types of diseases, take myocardial infarction for example, researchers discovered the over‐expression of S100A6 inhibited the cell apoptosis and significantly improved the infarct size *in vitro* ^11^. Meantime, S100A6 has been demonstrated to participate in numbers of signaling pathways like phosphoinositide 3‐kinase (PI3K)/protein kinase B (AKT), Wnt/β‐catenin, and nuclear factor‐κB ^12^. As a fundamental signaling pathway, the PI3K/AKT pathway is widely known that it participates in many cellular processes, including apoptosis, cell cycle, cell autophagy and cancer pathology ^13^, ^14^. Some studies revealed that maintaining the normal expression of PI3K/AKT pathway was able to maintain chondrocyte proliferation and produce anti‐apoptotic function^15^. Although much evidence has shown that S100A6 was involved in many biological activities, the specific mechanism of this molecular was still unclear.

According to recent research, there have been no studies to clarify the mechanism and function of S100A6 on osteoarthritis at a cellular level. Interestingly, we found that the expression of S100A6 was dramatically decreased in OA in our preliminary test. In order to further explore its mechanism in this study, we decided to create OA cell model *in vitro*. Though the pathological mechanism of OA is not fully understood, inflammatory responses are shown as a vital factor in the progress of OA. Interleukin‐1β (IL‐1β), as a promoter of many inflammatory factors, could be activated many inflammatory pathways to disturb the cartilage metabolism and was always used to create an OA model *in vitro* ^16^. In this study, we used rat chondrocytes as the research object, through virus transfection to regulate the expression of S100A6, then detected the characteristic inflammatory molecules of OA (IL‐6 & MMP‐13) and pathway node molecules (PI3K & AKT) and utilized Caspase‐3 activity and TUNEL assays to explore the mechanism of S100A6 in a cell model. We hypothesized S100A6 influenced the OA pathological process and utilized the PI3K/AKT pathway to regulate chondrocyte apoptosis. The objective of this study includes three points as below. First of all, we investigate the relationship between S100A6 and the pathological changes of osteoarthritis. Second, we need to confirm that S100A6 affected the chondrocytes apoptosis. Third, we explore if the influence triggered by S100A6 was produced by the regulatory of the PI3K/AKT signal pathway. Finally, our findings corroborate the experimental hypothesis and support that S100A6 is a potential therapeutic target for osteoarthritis.

## Materials and Methods

### Cell Culture and IL‐1β Induced OA Model

The rat cartilage chondrocytes (CP‐R092; Procell) were cultured in Dulbecco's modified Eagle's medium (Gibco) supplemented with 10% fetal bovine serum (Gibco) and 2% antibiotics (Penicillin and Streptomycin, Gibco). The cells were first revived from freezer stocks and then passaged in T175 flasks. The flasks were kept in an incubator at 37°C with 5% CO_2_. The cells were harvested when they had nearly reached confluence. While the OA model chondrocytes which were treated with IL‐1β (10 ng/mL) for 24 h were harvested for subsequent processes. PI3K/AKT pathway inhibitor LY294002 (10 μM) and pathway activator IGF‐1 (100 ng/mL) were incubated 24 h with some group of cells for relevant testing.

### In vitro Adenovirus Transfection was Used to Regulate the Expression of S100A6

Adenoviral vectors were designed and synthesized by Shanghai GenePharm Co., Ltd. The most efficient interference sequence was selected from three target sequences (S100a6‐Rat‐54, S100a6‐Rat‐153, S100a6‐Rat‐279). The small interference RNA was packaged in plasmid. The chondrocytes with good growth condition were inoculated and mixed with Lipofectamine™ 2000 (11668–019; Invitrogen, Thermo Fisher Scientific, Inc.) following the protocol. Cells were incubated for 24 h for the satisfactory expression of over‐expressed and down‐regulated S100A6 gene and cells transfected with empty vector were used as controls.

### Immunofluorescence Assay Identified the Expression of Collagen II

The expression of type II collagen was analyzed by immunofluorescence microscopy for identification of chondrocyte. The cells were fixed with 4% paraformaldehyde for 10 min after washing three times with PBS. Cells were subsequently blocked with goat blocking serum for 30 min. Then the samples were incubated with rabbit anti‐collagen II polyclonal antibody (ab34712; BACAM, 1:100 dilution) overnight at 4°C. After washing cells with PBST, the secondary antibody which was CY3 Conjugated AffiniPure goat Anti‐rabbit IgG (BA1032; Boster, 1:100 dilution) was added. Following incubation for 1 h at 37°C, the samples was counterstained with 4′,6‐diamidino‐2‐phenylindole (C1002; Beyotime) and finally observed with a fluorescence microscope (BX53; Olympus). Because collagen II is a characteristic protein produced by chondrocytes, the positive results were used to identify rat chondrocyte samples.

### Western blot analysis was taken to detect the expression pattern of S100A6, PI3K and AKT

Total proteins were prepared using TRIzol reagent following the protocol. Protein concentration was determined using a BCA Protein Assay Kit (P0010; Beyotime). Protein extracts were denatured in sample loading buffer and resolved by 12% SDS‐PAGE gel, and then the proteins were migrated from the gel onto the PVDF membrane (Immun‐BlotTM PVDF Membrane, Bio‐Rad) which was pre‐soaked in methanol and transfer buffer. The membranes were blocked with 5% nonfat dried milk in PBS for 1 h at room temperature and then incubated in blocking solution at 4°C overnight with primary antibodies, which included rabbit anti‐PI3K polyclonal antibody(Ab182651; BACAM, 1:500 dilution), rabbit anti‐AKT1 monoclonal antibody (Ab81283; BACAM, 1:1000 dilution) and rabbit anti‐S100 alpha 6 monoclonal antibody (Ab181975; BACAM, 1:1000 dilution). Membranes were incubated with a secondary antibody, which was HRP Conjugated AffiniPure goat Anti‐rabbit IgG (BA1054; Boster Biological Technology, Ltd., 1:5000 dilution) in blocking solution at room temperature for 1 h. Chemiluminescence was used to detect the target protein, the membranes were covered with substrate solution and incubated. The immunoreactive bands were quantified by using Image J software and GADPH was used as internal reference protein for quantitative analysis.

### RT‐PCR Assay Detected the Expression of S100A6, IL‐6, MMP13, PI3K and AKT

Total RNA from chondrocytes were extracted using TRIzol reagent (15596‐026; Ambion) and measured the absorbance at 260 and 280 nm for quantify. RT reactions were performed according to the protocol. RT‐PCR test was performed using SYBR Green PCR instrument on QuantStudio™ 6 Flex Real‐time PCR system (Thermo Fisher Scientific, Inc.). The cycling conditions were as follows: Initial denaturation at 95°C for 10 min, followed by 40 cycles of 15 s at 95°C, and 60 s at 60°C for annealing elongation. GADPH was used as the internal reference. The expression data were calculated using the 2^‐ΔΔCt^ method. All following primer sequences were used: *GAPDH*, 5′‐ACAGCAACAGGGTGGTGGAC‐3′ (forward), 5′‐ TTTGAGGGTGCAGCGAACTT‐3′ (reverse); *AKT*, 5′‐GCTCTTCTTCCACCTGTCTCG‐3′(forward), 5′‐ CACAGCCCGAAGTCCGTTA‐3′ (reverse); *PI3K*, 5′‐ GCAACAAGTCCTCTGCCAAA‐3′ (forward), 5′‐ ACGTAATAGAGGAGCTGGGC‐3′ (reverse); *IL‐6*, 5′‐ GTTGCCTTCTTGGGACTGATG‐3′ (forward), 5′‐ TACTGGTCTGTTGTGGGTGGT‐3′ (reverse); *MMP‐13*; 5′‐ ATACGAGCATCCATCCCGAG‐3′ (forward), 5′‐ CGTGTCCTCAAAGTGAACCG‐3′ (reverse); *S100A6*, 5′‐ CCCCCAGGAAGGCAACATAC‐3′ (forward), 5′‐ TGGCAAGGAGGGTGACAAGC‐3′ (reverse).

### Caspase‐3 Activity Assay Tested the Early Stage of Apoptosis Activity

The cells were harvested and assessed using Caspase‐3 activity assay kit (G015‐1; Nanjing Jiancheng Bioengineer Institute) following the standard protocol. The samples were lysed with lysis buffer (50 μL per 2 × 10^6^ cells) for 15 min on ice and centrifugation at 12000 rpm for 10 min. The equal amount of protein mixed with Ac‐DEVD‐pNA substrate were incubated at 37°C for 4 h. The values measured by colorimetric measurement of pNA at an absorbance of 405 nm. Through testing the amount of enzyme of caspase‐3 by cleavage of 1 nmol Ac‐DEVD‐pNA to produce 1 nmol pNA per hour, the caspase‐3 activity can be calculated according to the formula.

### TUNEL Staining Reflected the Late Stage of Apoptosis Activity

The TUNEL assay was applied using TUNEL BrightRed Apoptosis Detection Kit (A113‐03; Vazyme). According to the protocol, cells were fixed with 4% formaldehyde for 25 min and mixed with proteinase K solution for 20 min. Proper concentration of equilibration buffer was added in the samples within time. Then the samples were incubated with reaction mixture for 60 min and cell nuclei were stained with DAPI (C1002; Beyotime). After the samples were washed with PBS, the slides were observed under a fluorescence microscope using × 200 and × 400 times magnification. In order to analyze the data more intuitively, the apoptosis index which means the percentage of positive staining cells in the total number of cells in the microscopic field was calculated in each group.

### Main Outcome Measures

#### The Expression Level of S100A6

RT‐PCR showed the relative expression of mRNA. Compared with the reference gene, the calculated value showed the relative expression of target protein mRNA. Also, western blotting bands reflect the expression of the target protein, the color and width of the bands can represent the protein content. The expression of target molecular S100A6 were detected through transcription and translation levels separately, which illustrated its changes in osteoarthritis.

#### Inflammatory Biomarkers IL‐6 and MMP‐13

As the characteristic inflammatory molecules, IL‐6 and MMP‐13 were closely related to the destruction of chondrocyte and matrix, RT‐PCR was used to detect the expression of IL‐6 and MMP‐13, the values were proportional to the severity of osteoarthritis.

#### Caspase‐3 Activity

Caspase‐3 is the initiation molecule of caspase apoptosis pathway and its activity reflects the active degree of early apoptosis. The Caspase‐3 activity can be determined by pNA which is produced by a Caspase‐3 catalyzed substrate called Ac‐DEVD‐pNA (acetyl‐Asp‐Glu‐Val‐Asp p‐nitroanilide). The pNA could be quantified by measuring the optical density. Thus, the activity of Caspase‐3 can be calculated by the amount of pNA produced per unit time.

#### TUNEL (TdT‐Mediated dUTP Nick‐End Labeling) Stain and Apoptosis Index

In the late stage of apoptosis, DNA occurs fragmentation and exposes 3′‐OH terminal which can bind to fluorescein‐dUTP under the catalysis of terminal deoxynucleotidyl transferase (TdT). Fragmented DNA in the nucleus, which also represents apoptotic activity, can be observed by fluorescence staining. More intuitively, the intensity of apoptotic activity can be quantified by the percentage of positive staining cells in the total number of cells, which calculated as apoptosis index. The percentage was proportional to the activity of apoptosis.

#### The Expression Level of PI3K and AKT

PI3K and AKT are key node molecules which can reflect the status of PI3K/AKT signaling pathway, so their expression levels were detected by RT‐PCR and western blot and the value explained the up or down regulation of signaling pathway.

### Statistical Analysis

All data were analyzed with GraphPad Prism 8.0 software (GraphPad Software, Inc., San Diego, CA), and RStudio. The results were shown as the mean ± standard deviation. Student's t‐test and One‐way analysis of variance (ANOVA) were separately used to assess the comparison of two groups and three or more groups. A value of *P* < 0.05 was considered statistically significant.

## Results

### Chondrocyte Samples was Identified and S100A6 was Noticeably Down‐Expressed in OA Model

**Fig 1:**
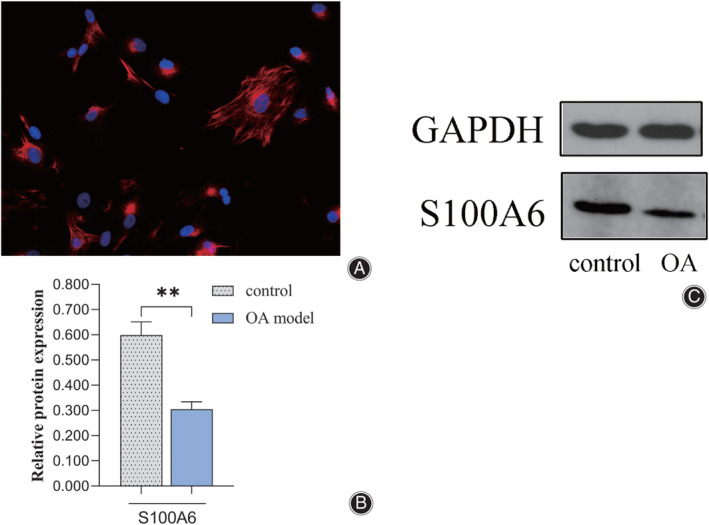
(A) Immunofluorescence assay revealed collagen II were labeled with red fluorescence and the nuclei of chondrocytes were labeled with blue fluorescence. The representative image is shown (×400 magnification). (B) Western blot assays showed the expression of S100A6 was obviously decreased in the OA model compared with the control group. Results are presented as mean ± SD, *P < 0.05, **P < 0.01, ***P < 0.001.

Immunofluorescence assay was performed to test type II collagen, which is produced by condrocyte and is one of the main components of cartilage. Under the fluorescence microscope (× 400 magnification), type II collagen were labeled red fluorescence and the nuclei of chondrocytes were labeled blue fluorescence (Fig. 1A). We detected the expression of S100A6 was dramatically reduced in the OA model compared with the control group by western blots (Fig. 1B,C), the relative expression level of the OA model and control group were 0.285 and 0.562 respectively, suggesting the significant correlation between S100A6 expression and osteoarthritis.

### S100A6 Improved Inflammatory Environment of OA by Down‐Regulated the Expression of IL‐6 and MMP‐13

**Fig 2:**
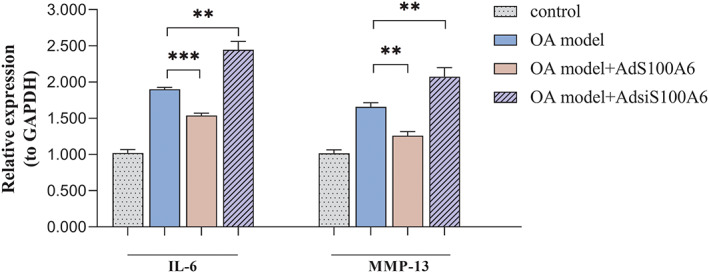
S100A6 affects the inflammation reaction of osteoarthritis. (A) The quantitative PCR assays showed the expression of IL‐6 and MMP‐13 were increased in in OA model. The values were decreased after the treatment of AdS100A6 and increased after the treatment of AdsiS100A6. (B, C) Western blot assays exhibited the expression of IL‐6 and MMP‐13 was increased in OA model. And the expression levels were down‐regulated by the treatment of AdS100A6 and up‐regulated by the treatment of AdsiS100A6. Results are presented as mean ± SD, *P < 0.05, **P < 0.01, ***P < 0.001.

To investigate the influence of S100A6 to inflammation, the mRNA level of matrix metalloproteinase‐13 (MMP‐13) and interleukin‐6 (IL‐6) were tested by RT‐PCR (Fig. 2). Following the treatment of adenovirus containing the S100A6 gene (AdS100A6), the expression level of mmp‐13 and IL‐6 were decreased apparently, compared with those of the OA group, the relative expression level was 1.259 and 1.534 respectively. However, accompanied by the S100A6, expression was down‐regulated by adenovirus contain S100A6 short interfering gene (AdsiS100A6), the expression of MMP‐13 and IL‐6 was significantly increased, the relative expression value was 2.074 and 2.445 respectively. Statistical analysis showed that these changes were significant. So, we revealed that the up‐regulation of S100A6 led to the decrease of MMP‐13 and IL‐6 expression, and down‐regulation of S100A6 induced the opposite influence. It is demonstrated the inflammation is affected by S100A6 in OA chondrocytes.

### Caspase‐3 Activity and TUNEL Staining Verified that S100A6 Mediated the Apoptosis of Chondrocytes

**Fig 3:**
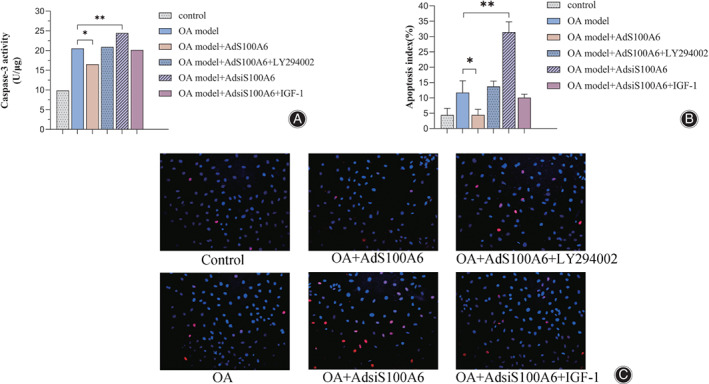
S100A6 mediates the apoptosis of chondrocytes. (A) Caspase‐3 activity assays showed the value was increased in the OA model. The caspase‐3 activity was decreased after the transfection of AdS100A6 and increased after the transfection of AdsiS100A6. The effects trigged by adenovirus transfection could be offset by using LY294002 and IGF‐1, respectively. (B) TUNEL assays exhibited the similar changes to caspase‐3 activity, which were quantified by the apoptosis index. (C) The TUNEL stain images showed the nuclei were labeled with blue fluorescence, and TUNEL positive cells were labeled with red fluorescence simultaneously (× 200 magnification). Results are presented as mean ± SD, *P < 0.05, **P < 0.01, ***P < 0.001.

In order to examine the effect of S100A6, a part of the OA chondrocytes was treated by adenovirus containing the S100A6 gene (AdS100A6) to up‐regulate the expression and some OA chondrocytes were transfected by adenovirus containing the S100A6 short interfering gene (AdsiS100A6) to down‐regulate the expression. First of all, the caspase‐3 activity assay shows the value of the OA model was nearly 2 fold compared with normal cells, the data were 20.549 U/μg and 9.881 U/μg. As presented in Fig. 3A, after the expression level of S100A6 was up‐regulated by AdS100A6, the value of caspase‐3 activity was 16.512 U/μg, which was decreased 0.2 fold compare with the OA group. Conversely, following the down‐regulation of S100A6 by AdsiS100A6, the condition of caspase‐3 activity was 24.450 U/μg, which was increased about 0.2 fold compared with the untreated OA group. Also, to further prove S100A6 was involved in the apoptosis of chondrocytes, the TUNEL stain was performed. Under the microscope (×200 magnification), we observed the nuclei were labeled with blue fluorescence, and TUNEL positive cells were labeled red fluorescence simultaneously (Fig. 3C). The apoptosis index, which means the percentage of positive cells in total cells in microscopic field, was calculated to measure the extent of apoptosis (Fig. 3B). The values shoed a dramatic difference between the control group and OA model group, the index was 4.42% and 11.21% respectively. The TUNEL positive cells were decreased after the treatment of AdS100A6, the index was 4.46%, which was close to the control group. Meanwhile if the OA group was treated with AdsiS100A6, the index was tripled compared with before, which was up to 31.44%. These results indicated that the S100A6 regulates the apoptosis of chondrocytes.

### S100A6 Regulated PI3K/AKT Signaling Pathway Through Affecting the Expression of PI3K and AKT and Thus Modified the Apoptosis

**Fig 4:**
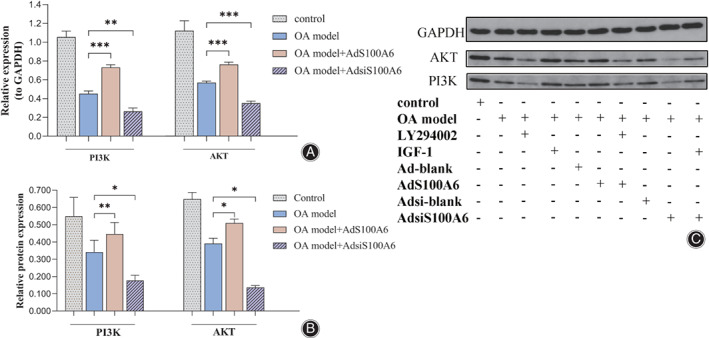
S100A6 regulated the PI3K/AKT signaling pathway. (A) The quantitative PCR assays showed the expression of PI3K and AKT raised after the treatment of AdS100A6 and reduced after the treatment of AdsiS100A6. (B, C) Western blot assays revealed the expression of p‐PI3K and p‐AKT were increased by the treatment of AdS100A6 and decreased by the treatment of AdsiS100A6. Results are presented as mean ± SD, *P < 0.05, **P < 0.01, ***P < 0.001.

To explore whether the PI3K/AKT pathway was changed by S100A6, the recombinant adenovirus was used to modify the expression of S100A6. Rat S100A6 gene (AdS100A6) and S100A6 short interfering RNA gene (AdsiS100A6) were transfected into the OA model chondrocytes respectively. RT‐PCR tests were performed to detect the mRNA level of PI3K and its downstream signal molecule AKT (Fig. 4A). The expression level of PI3K and AKT in the IL‐1β induced OA model were 0.452 and 0.569, which were nearly reduced 0.5‐fold compared with those of control group, Obviously, the expression level of PI3K and AKT were increased the in AdS100A6 group, which were 0.732 and 0.763 respectively. And the data were decreased in the AdsiS100A6 group, which were 0.264 and 0.351 respectively. Besides the transcription level, the protein translation condition was also analyzed in this study. Western blotting was performed to investigate the phosphorylation level of PI3K and AKT to further validate the conclusion (Fig. 4B, C). Similarly, comparing the OA group, the level of p‐PI3K and p‐AKT were raised in the AdS100A6 group and declined after AdsiS100A6 treatment with statistical significance. In summary, the expression of PI3K and AKT were changed resulting from S100A6 variation. Furthermore, due to the PI3K/AKT pathway being blocked by LY294002 (10 μM), the apoptotic activity attenuated by AdS100A6 was offset (Fig. 3A–C). The Caspase‐3 activity was 21.789 U/μg and the apoptosis index was 13.73%, which were very close to the value of the untreated OA group. When the pathway was activated by IGF‐1 (100 ng/mL), it significantly impaired the AdsiS100A6‐enhanced apoptosis (Fig. 3A–C). The Caspase‐3 activity was down to 18.916 U/μg and the apoptosis index was down to 10.09%. So, these results demonstrated that the usage of pathway inhibitor LY294002 and activator IGF‐1 measurably offset the impact of S100A6. It suggests that S100A6 mediates the chondrocytes apoptosis through the PI3K/AKT signaling pathway.

## Discussion

Osteoarthritis is characterized by the degeneration of cartilage, which is manifested as the abnormal apoptosis of chondrocyte and the loss of cartilage. At present, non‐surgical treatments of osteoarthritis include non‐drug therapy, such as exercise and weight control, medications like NSAIDs^17^, glucosamine or vitamin D^18^, ^19^, and intra‐articular injection therapy like hyaluronic acid^20^. All these methods can only relieve the clinical symptoms, none of them could fundamentally prevent pathological changes. The end‐stage patients inevitably suffer the complications of arthroplasty and financial burden. Thus, it is important to detect the potential biomarkers or novel therapy of osteoarthritis. According to present studies, S100A6 was involved in cell survival and apoptosis in many cancer diseases, such as breast cancer^21^, malignant thyroid neoplasm^22^, gastric cancer^10^. However, owing to the deficiency of relevant researches about S100A6 roles in osteoarthritis, its biological functions and research value remain to be fully clarified.

We detected the expression of S100A6 was decreased in OA model samples. To investigate the role of S100A6, small interference RNA was transfected into chondrocyte to modulate the expression of S100A6. Previous studies showed inflammatory reaction is associated closely with OA pathogenesis and symptems^23^. A wide range of studies demonstrated the pathogenic effect of interleukin‐6 (IL‐6) in OA, such as promoting protease expression, damaging cartilage matrix production^24^, ^25^. While the inhibition of IL‐6 expression was able to relieve the cartilage destruction in OA models^26^. Also, matrix metalloproteinase‐13(MMP‐13) was shown as a key proteolytic enzyme in the progression of OA. Through hydrolyzed type II collagen, which is the main component of extracellular matrix, MMP‐13 detrimentally affected the metabolic balance of cartilage and contributed in developing the degradation^27^, ^28^. To the best of our knowledge, no previous articles investigated the relationship between S100A6 and inflammation in OA. The results of our study showed the expression of IL‐6 and MMP‐13 were decreased by up‐regulating S100A6 in the mice OA model. While these two inflammatory factors expressed increased when S100A6 was down‐regulated. So, it was shown that S100A6 influenced the OA inflammatory response.

Additionally, metabolic molecules associated apoptosis were mitochondrial apoptotic pathway which represented with Bcl family and Caspase pathway. Caspase‐3 was an activator of the Caspase family, which initiated caspase apoptosis cascade^29^. As expected, the caspase‐3 assay showed when S100A6 was over expressed, the caspase‐3 activity was decreased apparently. In contrast, if the S100A6 expression was reduced by AdsiS100A6, the caspase‐3 activity was increased dramatically. Besides Caspase‐3 activity was used to reflect the early phase of cell apoptosis, the TUNEL stain was performed to test the late phase of apoptosis for further verify the results. Under the fluorescence microscope, the TUNEL‐positive cells (red fluorescence light) marked the condition of DNA fragmentation. Comparing the apoptosis index, the results were consistent with those of caspase‐3 assay.

A number of studies have showed the PI3K/AKT pathway is involved in cell proliferation, apoptosis, or even autophagy^30^. One of the aims of this study was to demonstrate whether the PI3K/AKT signaling pathway was involved in S100A6‐mediated apoptosis in OA chondrocytes. Under the circumstance in which the S100A6 expression was regulated by adenovirus transfection, the RT‐PCR and western blots results verified up‐regulated S100A6 raised the expression of PI3K and its downstream signaling modules AKT and down‐regulated S100A6 reduced those expression. In order to prove S100A6 produced the impact through the PI3K/AKT pathway, LY294002 and IGF‐1 were applied in the AdS100A6 group and AdsiS100A6 group respectively. The data showed the apoptosis triggered by S100A6 could be offset by PI3K/AKT pathway regulator (LY294002 and IGF‐1) to a large extent. Therefore, it was clear that S100A6 played a role in chondrocyte apoptosis, and this effect was related to the PI3K/AKT pathway.

In conclusion, this study showed the S100A6 was related to the inflammation of osteoarthritis. More importantly, this protein serves a key role in apoptosis of chondracytes by activated or impressed PI3K/AKT signaling pathway. So, these results improve the understanding of the pathophysiological process of osteoarthritis. And S100A6 may be used as a potential treatment target for patients. However, limitations still exist in this study. First, more *in vivo* researches are required to verify such issues. Also, more details on metabolic mechanism, take the downstream branch of PI3K/AKT pathway for example, are needed to be further studied. As a summary of this research, S100A6 may applied as a novel biomarker or medicine target in clinic.
